# Immunogenicity of sequential conjugate and non-conjugate pneumococcal vaccination in dialysis patients and kidney transplant recipients: a 12-month serological follow-up

**DOI:** 10.3389/fimmu.2026.1906893

**Published:** 2026-10-08

**Authors:** Sophie Novelli, Aimé Nun, Antoine Cases, Mathilde Bahuaud, Lucie Chevrier, Séverine Beaudreuil

**Affiliations:** 1 AP-HP, Hôpital Bicêtre, Service de Santé Publique, Le Kremlin-Bicêtre, France; 2 Université Paris-Saclay, UVSQ, Inserm, CESP, Le Kremlin-Bicêtre, France; 3 AP-HP-Centre Université de Paris, Hôpital Cochin, Service d’immunologie biologique, Paris, France; 4 Nephrology Dialysis Transplantation Department, Bicetre Hospital, University Hospital Paris Saclay, AP-HP, Le Kremlin-Bicêtre, France

**Keywords:** conjugate vaccine, dialysis, immune response, kidney transplantation, non-conjugate vaccine, pneumococcal vaccine

## Abstract

**Background:**

Pneumonia is the second most common infection in individuals with end-stage kidney disease and is associated with poor prognosis. The most frequently implicated pathogen is *Streptococcus pneumoniae*. Vaccine coverage is therefore essential. Different vaccination strategies have been implemented over time, depending on the period and availability of vaccines in each country.

**Aim:**

In a single-center observational study, we describe the serological response for 7 serotypes in dialysis patients (DP) and kidney transplant recipients (KTR) following a standard vaccination schedule against *Streptococcus pneumoniae*, consisting of one dose of the 13-valent conjugate vaccine (Prevenar 13^®^) followed 2 months later by one dose of the 23-valent non-conjugate vaccine (Pneumo 23^®^).

**Methods:**

We analyzed 61 participants (DP n=30, KTR n=31) who had completed the full vaccination schedule between January 2015 and December 2016. IgG titers against 7 pneumococcal serotypes (4, 6B, 9V, 14, 18C, 19F, 23F) were measured by ELISA before each vaccine dose and at 3, 6 and 12 months thereafter. We modeled changes in IgG levels over time. We estimated the probability of seroconversion and seroprotection (IgG titers > 1 µg/mL) for each serotype.

**Results:**

Overall, the immune response was stronger in DP than in KTR. IgG titers and seroconversion rates were higher in DP than in KTR, except for serotypes 6B, 19F and 23F. The cumulative probability of global response in KTR vs. DP was 32.3% vs. 62.2% at 3 months, 35.5% vs. 73.6% at 6 months and 39.3% vs. 73.6% at 12 months.

**Conclusion:**

The serological response to this pneumococcal vaccination schedule in individuals with end-stage kidney disease was modest overall but tended to be higher in DP than in KTR.

## Introduction

Pneumonia is the second most common infection among individuals with end-stage kidney disease (ESKD), after bloodstream infections, and is associated with high mortality and poor long-term prognosis (1–3).

*Streptococcus pneumoniae* is the most common pathogen causing community-acquired pneumonia. Pneumococcal immunization is recommended for patients with chronic kidney disease (CKD), particularly those treated with maintenance dialysis and kidney transplantation. Vaccination has been shown to reduce mortality in individuals with cardiac disease (4) and in those undergoing hemodialysis (5–8).

A vaccination strategy involving two types of pneumococcal vaccine is available against the most common pneumococcal serotypes: i) Pneumo 23^®^ (Sanofi), a non-conjugate polysaccharide vaccine covering 23 serotypes (PPSV23: 1, 2, 3, 4, 5, 6B, 7F, 9N, 9V, 10A, 11A, 12F, 14, 15B, 17F, 18C, 19A, 19F, 20, 22F, 23F, 33F), and ii) Prevenar 13^®^ (Pfizer), a protein-conjugate vaccine covering 13 serotypes (PCV13: 1, 3, 4, 5, 6A 6B, 7F, 9V, 18C, 19A, 19F, 14, 23F). Initially, only a single dose of PPSV23 was recommended. PPSV23 reduces the incidence of invasive pneumococcal disease (9); however, it is poorly immunogenic in young children, the elderly and immunocompromised individuals (10). Pneumococcal conjugate vaccines were then developed to improve pneumococcal vaccine immunogenicity. Previous French guidelines recommended a sequential vaccination schedule consisting of one dose of PCV13 followed 2 months later by one dose of PPSV23. Although this strategy has demonstrated efficacy in the general population, its effectiveness is reduced in immunocompromised patients, as reported, for example, in HIV patients (11), and remains poorly studied in individuals with ESKD. Moreover, no comparison of dialysis (hemodialysis or peritoneal dialysis) patients (DP) and kidney transplant recipients (KTR) is available. Vaccine response is expected to be reduced in KTR, as observed with the COVID-19 and influenza vaccines, due to immunosuppressive treatment (12, 13).

Newer conjugate vaccines covering 20 serotypes are now available. However, evaluating the previously recommended sequential schedule of PCV13 followed by PPSV23 offers valuable insights into the immune response in immunocompromised kidney populations and provides a useful context for interpreting the potential benefits of newer conjugate vaccines.

Below, we describe the serological response for seven serotypes in patients undergoing dialysis or living with a kidney transplant. The novelty of our study lies in the longitudinal, serotype-specific assessment, the comparison between DP and KTR, and the use of combined endpoints, including seroconversion and seroprotection, following the sequential PCV13 followed by PPSV23 schedule.

## Materials and methods

### Study population

This single-center prospective observational study was conducted between January 2015 and December 2016 as part of routine care in the Nephrology Department at Bicêtre Hospital, AP-HP Université Paris-Saclay, France. Eligible participants were adults aged ≥ 18 years who had been receiving hemodialysis or peritoneal dialysis for more than 3 months or had undergone kidney transplantation more than 6 months earlier, with no documented history of pneumococcal vaccination, based on patient report and/or the absence of vaccination records. Patients with an ongoing infection or other contraindication to vaccination were excluded. All eligible participants were included.

### Vaccination

As part of standard care, participants received an initial intramuscular dose of PCV13 (Prevenar 13^®^; Pfizer) at baseline, followed 2 months later by a single intramuscular dose of PPSV23 (Pneumo 23^®^; Sanofi), in accordance with the guidelines in effect at that time (14).

Participants attended monthly follow-up visits. Blood samples for serological assessment were collected before vaccination (month 0), immediately before PPSV23 administration (month 2), and at 3 months (approximately 1 month after PPSV23), 6 months and 12 months. Serological assessments were performed as part of routine clinical care and prospectively analyzed for this study.

### Serological assessment

All serological assessments were performed at the Immunology Laboratory of Hôpital Cochin, France. Immunoglobulin G (IgG) antibody concentrations for seven serotypes targeted by both PCV13 and PPSV23 (4, 6B, 9V, 14, 18C, 19F, 23F) were determined using a modified enzyme-linked immunosorbent assay. The seven serotypes were chosen for the reproducibility and robustness of the assay. In summary, 96-well plates (Corning, Inc., Corning, NY) were coated with a serotype-specific pneumococcal polysaccharide (PS) antigen (American Type Culture Collection, Manassas, VA) and incubated for 5 hours at 37 °C. Reference sera (007sp) and test specimens were pre-absorbed with 5 μg/ml pneumococcal C-polysaccharide (Statens Serum Institut, Copenhagen, Denmark) and 10 μg/ml serotype 22F capsular polysaccharide (American Type Culture Collection) for 30 minutes at room temperature before being serially diluted. After washing, serially diluted serum was added and the plates were incubated at room temperature for two hours. The plates were then washed again and alkaline phosphatase-conjugated goat anti-human IgG (Southern Biotech, Birmingham, AL) was added. Following a second two-hour incubation and washing, substrate (p-nitrophenyl phosphate in diethanolamine buffer, pH 9.8) was added. After final incubation, optical density was measured at 405 nm. Anti-pneumococcal antibody levels in each specimen were quantified by comparing linear regression plots with the reference serum (007sp) (National Institute for Biological Standards and Control [NIBSC]). The lower limits of quantification were 0.1 µg/mL for all serotypes, except serotype 14, for which the limit was 0.3 µg/mL. Any value below 0.1 was reported as < 0.1 µg/mL (< 0.3 for serotype 14). No upper limit of quantification was defined. Samples exceeding the quantifiable range were reanalyzed after additional dilution. Intra-assay variability ranged from 3% to 17.6%, depending on the serotype, while inter-assay variability ranged from 6.9% to 19%. All parameters were validated as part of the COFRAC accreditation process in 2018.

Samples from the same patient were analyzed over time, rather than all at once. Laboratory personnel were blinded to the clinical group.

### Ethics

All patients gave informed consent for vaccination. Pseudonymized routine care data were collected prospectively without any additional intervention beyond routine care. In accordance with French MR-004 requirements, patients were informed about the study and retained the right to opt out of the use of their data for research purposes.

### Data collection

Sociodemographic characteristics, clinical data and blood test results were collected from medical records. The Charlson’s score was calculated for each participant.

### Endpoints

The primary outcome was seroconversion and seroprotection for each of the seven serotypes. Based on studies defining criteria for pneumococcal vaccine immune response and on previous research in immunocompromised populations (15), we used the following definitions for seroconversion and seroprotection responses. Seroconversion was defined, for each serotype, as a two-fold increase from baseline in serotype-specific IgG concentration as measured by ELISA at any follow-up visit. Seroprotection was defined, for each serotype, as an IgG concentration ≥ 1 μg/mL. Seroresponse was defined as achieving both seroconversion and seroprotection for one serotype. Participants who achieved both seroconversion and seroprotection for at least five serotypes were classified as global responders.

### Statistical analysis

We first described the demographic and clinical characteristics of the participants, overall and by group: dialysis patients (DP) and kidney transplant recipients (KTR). Categorical variables were expressed as counts and percentages and compared using the Chi-squared test or Fisher’s exact test, as appropriate. Continuous variables were reported as medians with interquartile range and compared using the Mann-Whitney U test.

For each serotype, we modeled the decrease in log_10_-transformed IgG levels over time, using a piecewise linear mixed-effects model. Fixed effects included time since the first dose (in months), study group (KTR vs. DP), and the group-by-time interaction. A participant-specific random intercept was included to account for within-subject correlation. To quantify the effect of the second PPSV23 dose, we evaluated candidate breakpoints at months 2 and 3, corresponding to the timing of the second dose and the immediate post-dose period. These candidate breakpoints were selected based on clinical considerations and further supported by visual inspection of individual IgG trajectories, which suggested a change in slope within this interval. We compared single-breakpoint models at months 2 and 3 with a two-breakpoint model at months 2 and 3 to specifically assess whether PPSV23 administration was associated with a transient increase in IgG levels. The final model was selected as the best compromise between goodness of fit, assessed using the Akaike Information Criterion (AIC) and likelihood ratio test, the significance of the change in slope, and model parsimony.

In addition, we estimated the probability of seroconversion, seroprotection and global response using the Kaplan-Meier estimator, accounting for right-censoring due to incomplete follow-up. For each serotype, time to seroconversion was defined as the exact time (in days) from vaccination to the first serological assessment at which a ≥ 2-fold increase in IgG concentration relative to baseline was observed. Similarly, time to seroprotection was defined as the exact time (in days) at which IgG concentration reached ≥ 1 µg/mL. Time to global response was defined as the exact time (in days) at which a participant achieved both seroconversion and seroprotection for at least five of the seven evaluated serotypes. Cumulative probabilities of seroconversion, seroprotection or global response as time-to-event outcomes were estimated at 3, 6 and 12 months. Participants who did not experience the relevant event were censored at their last available follow-up visit. Participants who already met the seroprotection criterion at baseline were assigned an event time of zero. Kaplan-Meier curves were compared between DP and KTR using the log-rank test. Because immunogenicity outcomes were assessed only at scheduled follow-up visits, the exact time of event occurrence was interval-censored: for each participant, the event was known to have occurred between the last visit without the event (left endpoint) and the first visit with the event (right endpoint), while participants without an observed event by their last visit were treated as right-censored. Therefore, as a sensitivity analysis, time to seroconversion was additionally analyzed using the nonparametric Turnbull estimator for interval-censored data, and DP and KTR groups were compared using a generalized log-rank test for interval-censored data.

To identify factors associated with global response, Cox proportional hazards regression models were fitted using time to global response as the outcome. In univariable analyses, we evaluated the following variables: study group (KTR vs. DP), age, sex, body mass index and diabetes. Variables associated with the outcome in univariable analyses or considered clinically relevant were then included in the multivariable model. The final multivariable model retained kidney transplant status, age, and body mass index. Hazard ratios (HRs) and 95% confidence intervals (CIs) were reported. The proportional hazards assumption was evaluated using statistical tests and graphical diagnostics based on scaled Schoenfeld residuals. As the proportional hazards assumption was violated for KTR versus DP status (Schoenfeld residuals test, p = 0.006), a time-varying coefficient model was fitted with a cutpoint at month 3 based on the smoothed Schoenfeld residuals plot. The linearity of age as a continuous predictor was assessed by comparing models with age treated as either a continuous or categorized variable using the Akaike Information Criterion (AIC). The assumption of linearity was not violated.

Among participants classified as global responders, we further characterized the quality of the immune response by calculating, for each serotype, the proportion of participants who achieved both seroconversion and seroprotection. Analyses were restricted to participants who had completed the full vaccination schedule and for whom data were available for at least two follow-up time points. Missing data were not imputed. All analyses were performed using R Statistical Software (v4.1.1; R Core Team 2021).

## Results

### Baseline characteristics

Between January 2015 and December 2016, 80 consecutive eligible adults were enrolled in the study. Of these, 61 had completed the full vaccination schedule and had available serological measurements both before and after vaccination (**Supplementary Figure 1**). 19 participants were ultimately excluded: 12 had no follow-up visit after the first dose, 6 received the second dose > 3 months after the first, and 1 had no serological data at baseline. Baseline characteristics were also compared between included and excluded participants. A higher proportion of DP was observed among those included in the study than among those excluded (31/61 [51%] vs. 4/19 [21%]), whereas no differences were observed for age, sex, BMI, or the prevalence of diabetes or cancer. Among the 61 participants analyzed, 30 were DP (9 on peritoneal dialysis and 21 on hemodialysis), and 31 were KTR. Their median age was 59 (IQR, 48-64), and 74% were male. 10 patients had a history of cancer: skin tumors (n=2) treated by surgical excision only, kidney cancer (n=3) treated by nephrectomy only, multiple myeloma (n=1) in remission for 5 years, colon cancer (n=1) treated by colectomy 10 years ago, breast cancer (n=1) in remission for more than 20 years, *in situ* bladder tumor (n=1) for more than 10 years, and cervical cancer (n=1) in remission for more than 10 years.

Baseline characteristics are shown in (**Table 1**). No statistically significant difference was observed between the DP and KTR groups, except for hemoglobin and albumin levels, which were higher in KTR.

**Table 1:** Participant characteristics.

Characteristic	Overall, N = 61	Dialysis, N = 30	Transplant, N = 31	P-value^1^
Sex				0.94
Female	16 (26%)	8 (27%)	8 (26%)	
Male	45 (74%)	22 (73%)	23 (74%)	
Age, years	59 (48, 64)	58 (45, 64)	61 (49, 64)	0.50
18 – 40	7 (11%)	6 (20%)	1 (3.2%)	
41 – 60	26 (43%)	12 (40%)	14 (45%)	
61 – 80	26 (43%)	10 (33%)	16 (52%)	
> 80	2 (3.3%)	2 (6.7%)	0 (0%)	
Body mass index	23.9 (22.0, 27.3)	24.1 (21.5, 27.2)	23.7 (22.1, 27.4)	0.76
Normal (18.5 – 25)	34 (57%)	18 (62%)	16 (52%)	
Underweight (< 18.5)	2 (3.3%)	0 (0%)	2 (6.5%)	
Obese (> 30)	7 (12%)	2 (6.9%)	5 (16%)	
Overweight (25 – 30)	17 (28%)	9 (31%)	8 (26%)	
Charlson’s score	5.0 (3.0, 7.0)	5.0 (2.3, 7.8)	4.0 (3.0, 7.0)	0.76
Nephropathy				0.61
Diabetes	8 (13%)	4 (13%)	4 (13%)	
Glomerulopathy	14 (23%)	8 (27%)	6 (19%)	
Undetermined	11 (18%)	5 (17%)	6 (19%)	
Myeloma	1 (1.6%)	0 (0%)	1 (3.2%)	
Chronic interstitial nephritis	2 (3.3%)	2 (6.7%)	0 (0%)	
Polycystic kidney disease	5 (8.2%)	1 (3.3%)	4 (13%)	
Uropathy	6 (9.8%)	4 (13%)	2 (6.5%)	
Vascular nephropathy	14 (23%)	6 (20%)	8 (26%)	
Cancer	10 (16%)	3 (10%)	7 (23%)	0.30
Diabetes	20 (33%)	8 (27%)	12 (39%)	0.32
Dialysis duration, months		9.3 (4.7, 19)	Not applicable	
Time since transplantation, months		Not applicable	3.2 (1.7, 10.2)	
Immunosuppressive regimen				
Belatacept/mycophenolate ofetil/prednisone			24 (77%)	
Tacrolimus/mycophenolate ofetil/prednisone			4 (13%)	
Everolimus/mycophenolate mofetil/prednisone			1 (3.2%)	
Mycophenolate mofetil/prednisone			1 (3.2%)	
Creatinine		Not applicable	143 (120, 180)	
Hemoglobin	12.0 (10.8, 13.0)	11.0 (10.1, 12.1)	13.0 (12.0, 13.7)	< 0.001
Leucocytes	6.3 (4.9, 7.7)	6.3 (4.9, 7.7)	6.3 (5.1, 7.7)	0.92
Lymphocytes	1.1 (0.9, 1.7)	1.2 (1.0, 1.9)	1.1 (0.9, 1.6)	0.10
CD4 (/µl)	420 (309, 584)	517 (357, 663)	349 (266, 501)	0.04
CD8 (/µl)	356 (253, 520)	346 (243, 527)	375 (277, 513)	0.53
Platelets	193 (158, 242)	183 (155, 230)	198 (161, 243)	0.66
Albuminemia	40.0 (37.0, 43.0)	37.5 (34.3, 40.0)	42.0 (39.0, 43.0)	< 0.001
IgG (g/l)	9.6 (7.6, 11.3)	10.5 (7.6, 12.7)	9.3 (7.1, 10.7)	
IgM (g/l)	0.7 (0.5, 0.9)	0.7 (0.5, 0.9)	0.7 (0.5, 0.8)	
IgA (g/l)	1.7 (1.2, 3.1)	2.3 (1.5, 3.5)	1.4 (1.19, 2.3)	

Results are shown in n (%) or median (IQR).

^1^ Pearson’s Chi-squared test; Mann-Whitney U test.

In KTR, the maintenance immunosuppressive regimens included belatacept, mycophenolate mofetil and steroids in 77% of participants (n=24), and tacrolimus, mycophenolate mofetil and steroids in 13% (n=4). The remaining participants were receiving either everolimus and steroids, or everolimus, mycophenolate mofetil and steroids (**Table 1**).

### Immune response

#### Baseline antibody levels

At baseline, between 10% (serotype 4) and 74.2% (serotype 14) of participants had IgG levels above the protective threshold of ≥ 1 µg/mL prior to the first PCV13 dose, with no statistically significant difference in IgG titers between KTR and DP (**Table 2**).

Table 2Changes in IgG titers in dialysis and transplant participants.

**Table:** 

Endpoint	Serotype 4		Serotype 6B			Serotype 9V		Serotype 14
Baseline	Dialysis	Kidney transplant	Dialysis	Kidney transplant	Dialysis	Kidney transplant	Dialysis	Kidney transplant
Geometric mean titer (95% CI)	0.3 (0.2 – 0.5)	0.3 (0.2 – 0.4)	0.7 (0.4 – 1.2)	0.9 (0.6 – 1.5)	0.7 (0.4 – 1.0)	0.6 (0.4 – 0.9)	2.1 (1.2 – 3.6)	2.5 (1.5 – 4.3)
Seroprotection rate % (95% CI)	10.0 (0.0 – 20.1)	12.9 (0.3 – 23.9)	37.9 (17.5 – 53.3)	48.4 (27.4 – 63.3)	30.0 (11.5 – 44.6)	32.3 (13.6 – 46.9)	69.0 (46.6 – 82.0)	74.2 (53.1 – 85.8)
3 months								
Geometric mean titer (95% CI)	1.8 (1.2 – 2.7)	0.9 (0.6 – 1.3)	5.0 (3.1 – 8.1)	3.1 (2.0 – 5.0)	3.0 (2.0 – 4.6)	1.6 (1.1 – 2.5)	15.2 (9.2 – 25.4)	6.8 (4.1 – 11.2)
Seroprotection rate % (95% CI)	78.0 (56.1 – 89.0)	51.6 (30.4 – 66.4)	70.3 (47.4 – 83.2)	64.5 (43.0 – 77.9)	67.9 (45.4 – 81.1)	64.5 (43.0 – 77.9)	70.1 (47.2 – 83.1)	58.1 (36.5 – 72.3)
Seroconversion rate % (95% CI)	74.5 (52.4 – 86.4)	39.3 (19.2 – 54.4)	82.8 (61.7 – 92.2)	80.6 (60.3 – 90.6)	84.8 (63.5 – 93.6)	67.7 (46.3 – 80.6)	89.7 (69.8 – 96.5)	90.3 (71.6 – 96.7)
Seroresponse rate % (95% CI)	67.7 (45.3 – 80.9)	32.9 (13.9 – 47.7)	63.2 (40.2 – 77.4)	54.8 (33.4 – 69.4)	62.2 (39.2 – 76.5)	48.4 (27.4 – 63.3)	70.1 (47.2 – 83.1)	48.4 (27.4 – 63.3)
6 months								
Geometric mean titer (95% CI)	1.6 (1.0 – 2.4)	0.8 (0.5 – 1.2)	4.3 (2.7 – 6.9)	2.7 (1.7 – 4.3)	2.8 (1.8 – 4.2)	1.4 (0.9 – 2.2)	14.6 (8.8 – 24.2)	6.4 (3.9 – 10.5)
Seroprotection rate % (95% CI)	81.7 (60.1 – 91.6)	61.3 (39.7 – 75.1)	81.4 (59.3 – 91.5)	71.0 (49.7 – 83.3)	85.7 (64.8 – 94.2)	64.5 (43.0 – 77.9)	82.9 (59.8 – 92.8)	58.1 (36.5 – 72.3)
Seroconversion rate % (95% CI)	85.5 (64.4 – 94.1)	39.3 (19.2 – 54.4)	87.1 (65.6 – 95.1)	87.1 (67.8 – 94.8)	84.8 (63.5 – 93.6)	71.0 (49.7 – 83.3)	94.8 (70.2 – 99.1)	93.5 (75.3 – 98.3)
Seroresponse rate % (95% CI)	78.5 (56.6 – 89.3)	36.3 (16.6 – 51.3)	71.4 (48 – 84.3)	64.5 (43 – 77.9)	77.3 (54.6 – 88.7)	51.6 (30.4 – 66.4)	82.9 (59.8 – 92.8)	51.6 (30.4 – 66.4)
12 months								
Geometric mean titer (95% CI)	1.2 (0.7 – 2.1)	0.6 (0.4 – 1.0)	3.2 (1.8 – 5.7)	2.1 (1.2 – 3.5)	2.3 (1.4 – 3.8)	1.1 (0.7 – 1.7)	13.5 (7.5 – 24.3)	5.7 (3.3 – 9.8)
Seroprotection rate % (95% CI)	81.7 (60.1 – 91.6)	68.3 (46.6 – 81.2)	81.4 (59.3 – 91.5)	75.1 (53.4 – 86.7)	85.7 (64.8 – 94.2)	67.7 (46.3 – 80.6)	88.6 (63.2 – 96.5)	58.1 (36.5 – 72.3)
Seroconversion rate % (95% CI)	85.5 (64.4 – 94.1)	39.3 (19.2 – 54.4)	87.1 (65.6 – 95.1)	87.1 (67.8 – 94.8)	84.8 (63.5 – 93.6)	71.0 (49.7 – 83.3)	100	93.5 (75.3 – 98.3)
Seroresponse rate % (95% CI)	78.5 (56.6 – 89.3)	36.3 (16.6 – 51.3)	71.4 (48 – 84.3)	68.5 (46.5 – 81.4)	77.3 (54.6 – 88.7)	51.6 (30.4 – 66.4)	88.6 (63.2 – 96.5)	51.6 (30.4 – 66.4)

**Table:** 

Endpoint	Serotype 18C		Serotype 19F		Serotype 23F	
Baseline	Dialysis	Kidney transplant	Dialysis	Kidney transplant	Dialysis	Kidney transplant
Geometric mean titer (95% CI)	0.8 (0.6 – 1.3)	0.8 (0.6 – 1.2)	1.0 (0.6 – 1.6)	1.3 (0.8 – 2.0)	0.5 (0.2 – 0.9)	0.6 (0.3 – 1.2)
Seroprotection rate % (95% CI)	40.0 (19.6 – 55.2)	45.2 (24.5 – 60.2)	43.3 (22.5 – 58.6)	61.3 (39.7 – 75.1)	26.7 (9.0 – 40.9)	29.0 (11.1 – 43.3)
3 months						
Geometric mean titer (95% CI)	5.0 (3.5 – 7.3)	2.6 (1.8 – 3.7)	4.2 (2.6 – 6.6)	4.1 (2.6 – 6.4)	3.5 (1.9 – 6.5)	2.1 (1.2 – 3.9)
Seroprotection rate % (95% CI)	65.0 (42.1 – 78.8)	54.8 (33.4 – 69.4)	61.7 (38.9 – 76)	48.4 (27.4 – 63.3)	69.2 (46.2 – 82.4)	52.7 (31.0 – 67.5)
Seroconversion rate % (95% CI)	80.0 (59.1 – 90.2)	77.4 (56.7 – 88.2)	77.5 (55.7 – 88.6)	93.5 (75.3 – 98.3)	71.5 (48.8 – 84.1)	54.8 (33.4 – 69.4)
Seroresponse rate % (95% CI)	61.5 (38.7 – 75.9)	41.9 (21.7 – 56.9)	54.8 (32.3 – 69.8)	45.2 (24.5 – 60.2)	65.7 (42.7 – 79.5)	39.5 (19.3 – 54.7)
6 months						
Geometric mean titer (95% CI)	4.3 (3.0 – 6.3)	2.3 (1.6 – 3.3)	3.7 (2.3 – 5.8)	3.5 (2.3 – 5.5)	3.1 (1.7 – 5.6)	1.8 (1.0 – 3.3)
Seroprotection rate % (95% CI)	72.8 (49.8 – 85.2)	58.1 (36.5 – 72.3)	80.9 (58.4 – 91.2)	54.8 (33.4 – 69.4)	80.8 (58.3 – 91.1)	59.4 (37.4 – 73.7)
Seroconversion rate % (95% CI)	93.3 (74.6 – 98.3)	83.9 (64.0 – 92.8)	88.8 (67.9 – 96.1)	93.5 (75.3 – 98.3)	75.6 (52.7 – 87.4)	58.1 (36.5 – 72.3)
Seroresponse rate % (95% CI)	73.1 (50.1 – 85.5)	48.4 (27.4 – 63.3)	77.4 (54.7 – 88.7)	51.6 (30.4 – 66.4)	73.3 (50.3 – 85.7)	46.2 (25.1 – 61.4)
12 months						
Geometric mean titer (95% CI)	3.2 (2.0 – 5.2)	1.8 (1.2 – 2.8)	2.9 (1.6 – 4.9)	2.6 (1.6 – 4.3)	2.4 (1.2 – 4.8)	1.4 (0.7 – 2.6)
Seroprotection rate % (95% CI)	72.8 (49.8 – 85.2)	61.6 (39.8 – 75.4)	80.9 (58.4 – 91.2)	62.1 (40.1 – 76)	80.8 (58.3 – 91.1)	63.1 (40.9 – 77)
Seroconversion rate % (95% CI)	93.3 (74.6 – 98.3)	83.9 (64.0 – 92.8)	88.8 (67.9 – 96.1)	93.5 (75.3 – 98.3)	75.6 (52.7 – 87.4)	62.3 (40.0 – 76.2)
Seroresponse rate % (95% CI)	73.1 (50.1 – 85.5)	52.1 (30.6 – 66.9)	77.4 (54.7 – 88.7)	58.8 (36.9 – 73.1)	73.3 (50.3 – 85.7)	50.4 (28.4 – 65.6)

CI, confidence interval. Seroprotection: IgG titers ≥ 1 µg/mL; seroconversion: ≥ 2-fold increase in IgG titers after vaccination. Probabilities of seroprotection and seroconversion were estimated using the Kaplan-Meier estimator. Participants with baseline IgG titers ≥ 1 µg/mL were considered seroprotected at time zero in the Kaplan-Meier analysis. Geometric mean titers were estimated using a linear mixed-effects model. Seroresponse was defined as achieving both seroconversion and seroprotection for a given serotype. Time is from the first PCV13 dose. 3 months corresponds to approximately 1 month after PPSV23. Number of participants: dialysis, n = 30; kidney transplant, n = 31.

#### IgG titers kinetics

The modeling of changes in IgG titers is presented in **Figure 1**. The best-fitting model included a significant change in slope at month 2, corresponding to the timing of the second PPSV23 dose (**Supplementary Table 1**). For all serotypes, the first PCV13 dose induced a significant increase in IgG levels over the first 2 months, followed by a gradual decline. To address the effect of the second PPSV23 dose, we tested for a potential rebound or additional increase in IgG titers between months 2 and 3. Adding a second breakpoint at month 3 (approximately 1 month after PPSV23) did not improve model fit (non-significant likelihood ratio test). Furthermore, the slope between months 2 and 3 was not significantly different from zero, providing no evidence of a rebound or additional increase in IgG titers after the second PPSV23 dose for any serotype or study group. KTR exhibited significantly lower IgG titers than DP over the 12-month follow-up period for more than half of the serotypes (4, 9V, 14, 18C). No statistically significant differences were observed for serotypes 6B, 19F and 23F.

**Figure 1:**
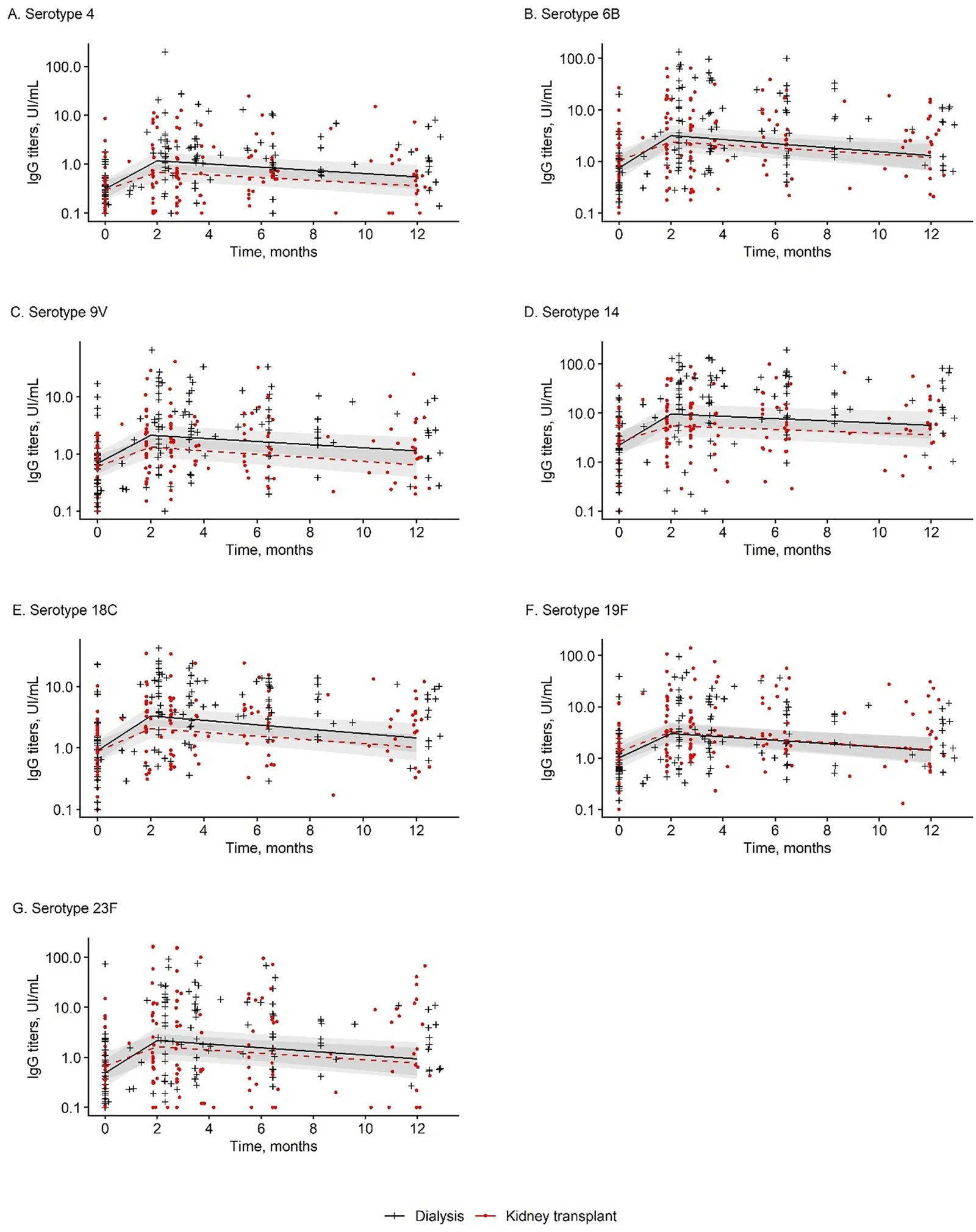
Kinetics of serotype-specific IgG concentrations in dialysis and transplant participants for pneumococcal serotypes **(A)** 4, **(B)** 6B, **(C)** 9V, **(D)** 14, **(E)** 1 8C, **(F)** 19F and **(G)** 23F. IgG concentrations are shown on a logarithmic scale (log_10_-transformed axis). Time in months is from the first PCV13 dose. Circles (red) and crosses (black) represent observed circulating IgG antibody levels in dialysis patients (DP) and kidney transplant recipients (KTR), respectively. Dashed (red) and solid (black) lines indicate predicted mean IgG trajectories estimated using a piecewise linear mixed-effects model for DP and KTR, respectively. Shaded areas represent the 95% confidence intervals around the predicted mean values.

#### Seroconversion

Seroconversion rates for each serotype are shown in **Table 2**. Most participants achieved a two-fold increase in IgG levels. Among DP, seroconversion rates at 6 months (approximately 4 months after PPSV23) ranged from 75.6% to 94.8%, depending on the serotype. In comparison, seroconversion rates in KTR were lower for most serotypes, ranging from 56% to 71%, although the differences were not statistically significant (log-rank test). As a sensitivity analysis, time to seroconversion was analyzed using the Turnbull estimator, which showed a similar overall pattern as the primary analysis. The generalized log-rank test for interval-censored data did not identify significant differences between groups (**Supplementary Figure 2**).

#### Seroprotection

Similarly, among participants with baseline IgG levels < 1 µg/mL, seroprotection rates were higher in DP than in KTR throughout the study period (**Table 2**).

#### Global response

Participants who achieved both seroconversion and seroprotection for at least five serotypes were classified as global responders. The cumulative probability of global response was 62.2%, 73.6% and 73.6% in DP at 3, 6 and 12 months, respectively, versus 32.3%, 35.5% and 39.3% in KTR (**Table 3**).

**Table 3:** Percentages (95% confidence interval) of global responders.

Months	Dialysis (n = 30)	Kidney transplant (n = 31)
3	62.2 (39.2 – 76.5)	32.3 (13.6 – 46.9)
6	73.6 (50.6 – 85.8)	35.5 (16.2 – 50.3)
12	73.6 (50.6 – 85.8)	39.3 (19.1 – 54.4)

Participants who achieved both seroconversion and seroprotection for at least five serotypes were classified as global responders. Percentages were estimated using the Kaplan-Meier estimator. Time is from the first PCV13 dose. 3 months corresponds to approximately 1 month after PPSV23.

Factors associated with global response were assessed using univariable and multivariable Cox models (**Supplementary Table 2**). As the proportional hazards assumption was violated for KTR versus DP status (Schoenfeld residuals test, p = 0.006), the effect of kidney transplantation versus dialysis was modeled using a time-varying coefficient, with a prespecified cutpoint at month 3 based on the smoothed Schoenfeld residuals plot. In univariable analysis, KTR showed a non-significant trend toward a lower probability of global response compared with DP during the first 3 months (HR = 0.51, 95% CI: 0.23-1.11, p = 0.09) and after beyond 3 months (HR = 0.27; 95% CI, 0.05-1.62; p = 0.15). In multivariable analysis, the association between transplant status and global response remained non-significant during the first 3 months (HR = 0.53, 95% CI: 0.23-1.17, p = 0.11) and beyond 3 months (HR = 0.18; 95% CI, 0.02-1.15; p = 0.07), with the latter association approaching statistical significance. Abnormal BMI (vs. normal, 18.5–25 kg/m²) was associated with a lower probability of global response in both univariable and multivariable analysis (adjusted HR = 0.36; 95% CI, 0.16-0.80; p = 0.01). We further characterized the quality of the immune response among participants who achieved a global response. Among global responders, we assessed the proportion who achieved both seroprotection and seroconversion for each serotype. DP had a higher proportion of combined seroprotection and seroconversion than KTR for serotypes 4 and 23F (both p = 0.05) (**Table 4**).

**Table 4:** Percentage of global responders achieving both seroconversion and seroprotection for each serotype.

Global responders				
Serotypes	Overall (n = 33)	Dialysis (n = 21)	Kidney transplant (n = 12)	P-value*
4	84.8% (28)	95.2% (20)	66.7% (8)	0.05
6B^a^	93.8% (30)	90.0% (18)	100.0% (12)	0.50
9V	97.0% (32)	95.2% (20)	100.0% (12)	0.90
14^a^	90.6% (29)	95.0% (19)	83.3% (10)	0.50
18C	81.8% (27)	85.7% (18)	75.0% (9)	0.60
19F	97.0% (32)	95.2% (20)	100.0% (12)	0.90
23F	87.9% (29)	100.0% (21)	66.7% (8)	0.01

Values represent the percentage of global responders with both seroconversion and seroprotection for the indicated serotype. The denominator is the number of global responders in each group.

^a^ one missing value for a dialyzed participant.

* Fisher’s exact test < 0.05.

## Discussion

To our knowledge, this is among the first studies to report response for seven serotypes in DP and KTR, including the rates of seroconversion, seroprotection and global response, following a pneumococcal vaccination schedule consisting of one dose of PCV13 (Prevenar 13^®^) followed 2 months later by one dose of PPSV23 (Pneumo 23^®^), with a 12-month follow-up.

We observed that IgG titers for each serotype were higher after vaccination than before vaccination in both patient groups at all time points over the 12-month follow-up. However, the second PPSV23 dose did not appear to produce a rebound or additional increase in IgG titers. Seroconversion rates in KTR were lower for most serotypes, ranging from 56% to 71%, although the differences were not statistically significant. Similarly, among participants with baseline IgG levels < 1 µg/mL, seroprotection rates were higher in DP than in KTR throughout the study period.

The proportion of global responders was relatively low overall, and lower in KTR than in DP. Fewer than 40% of KTR achieved both seroprotection and seroconversion for at least five serotypes. Most KTR in our study were treated with belatacept, which is known to induce a weak serological response after vaccination, including notably after the COVID-19 vaccine (12, 13). Given the limited sample size, we could not assess vaccine response according to specific immunosuppressive strategies, and the generalizability of our findings to KTR receiving other immunosuppressive regimens remains uncertain.

Serological response after pneumococcal vaccination has been poorly studied in patients with CKD, and the available results are heterogeneous. However, studying serological response after vaccination is important, because the incidence of disease-causing serotypes varies over time and reflects the vaccination strategies implemented in adults and children in each country, which may affect the generalizability and clinical implications of serotype-specific findings (16). In a previous study, only 21% of patients with CKD stages 1 to 4 showed a serological response after one dose of PPSV23 (17). To date, only one study has reported the serological response in DP following a pneumococcal vaccination schedule consisting of one dose of PCV13 followed 2 months later by one dose of PPSV23. That study compared two vaccination strategies: a single dose of PPSV23 (Pneumo 23^®^) versus a sequential schedule of one dose of PCV13 (Prevenar 13^®^) followed 2 months later by one dose of PPSV23 (Pneumo 23^®^). No differences were observed in the mean antibody titers between the two strategies. Moreover, antibody titers were significantly lower at 6 months than at 1 month. The study did not report titers for individual serotypes, nor did it describe seroprotection or seroconversion rates (18).

Other studies have reported the serological response following a single dose of either PCV13 or PPSV23. In one study, the authors compared the serological response in patients that received either a single PCV13 or PPSV23 dose during dialysis and a single PCV13 dose after kidney transplantation. Immunogenicity was higher with PCV13 than PPSV23 in DP, and revaccination with PCV13 was both immunogenic and safe in KTR (19). In a second study including 51 patients (n=14 DP, n=37 KTR), the serological response after a single PPSV23 dose was positive in 99% of patients at 1 month, 94% at 6 months, and 85% at 12 months (20). In that study, KTR exhibited higher IgG2 levels than DP at 12 months. However, no differences were observed between the groups in total IgG levels. Serological response was defined based on the mean IgG and IgG2 levels, without distinguishing antibody levels for individual serotypes or reporting seroconversion or seroprotection (20).

Lastly, a study analyzed 155 DP divided into four groups based on vaccination status. PPSV23-naive patients were randomized to receive a single dose of either PPSV23 or PCV13, whereas PPSV23-pre-vaccinated patients, categorized according to whether vaccination had occurred less than or more than 4 years before study enrollment, all received a single dose of PCV13. In PPSV23-pre-vaccinated patients, immune responses to both PPSV23 and PCV13 remained significant for at least 1 year. Among vaccine-naive DP, PCV13 appeared to be more immunogenic than PPSV23; however, the response to PCV13 was weaker in PPSV23-pre-vaccinated patients than in vaccine-naive patients (21).

The impact of different vaccination strategies on morbidity and mortality among DP remains debated. Most available studies are observational and may be subject to healthy-vaccinee bias and confounding.

In a recent French study, a reduction in mortality was obtained with both PCV13 followed 2 months later by PPSV23 and with PCV13 alone (HR = 0.37; 95% CI, [0.28-0.51]; and HR = 0.35; 95% CI, [0.19-0.22]; p = 0.65, respectively), but not with PPSV23 alone (5). These findings contrast with those of a retrospective study of 160 DP, which found, after propensity score matching, that PPSV23 alone was associated with higher survival than no vaccination, whereas no difference in survival was observed among patients who received PCV13 followed 2 months later by PPSV23 (6).

Recently, a new 20-valent conjugate vaccine (Prevenar 20^®^; PCV20), targeting the 13 serotypes included in PCV13 plus serotypes 8, 10A, 11A, 12F, 15B/C, 22F and 33F, has been developed, with only a single dose required. It has been shown to be safe and effective in the general population (22). Evaluating its use in DP and KTR would be of interest, as a single-dose regimen could potentially improve vaccination coverage, which currently stands at only 22.3% in France (5). Single-dose, broader-valency conjugate vaccines may be attractive because completion of a two-dose schedule can be challenging and the present data suggest limited additional boosting after PPSV23.

Our study has several limitations. First, the small sample size, single-center design, exclusion of 19 of 80 initially enrolled participants, and 1-year follow-up precluded comparisons between patients receiving hemodialysis and those receiving peritoneal dialysis. Second, we were unable to assess the association between the occurrence of infections and the serological response to vaccination. We did not assess pneumonia, invasive pneumococcal disease, hospitalization, or mortality. Third, we did not include comparison groups receiving PCV13 alone, PPSV23 alone, or newer conjugate vaccines such as PCV20.

Moreover, we did not evaluate the impact of kidney transplantation on the serological response in DP who had been vaccinated before transplantation. Most KTR recipients in our study were treated with belatacept.

Baseline seroprotection varied substantially across serotypes, likely reflecting differences in prior natural exposure to pneumococci or undocumented previous vaccination. Participants with high baseline IgG concentrations may have been less likely to achieve the predefined ≥ 2-fold increase following vaccination despite already having protective antibody levels, potentially resulting in an underestimation of the vaccine response. Our study did not assess opsonophagocytic activity or other measures of functional antibody activity. Therefore, although serotype-specific IgG concentrations are an established measure of vaccine immunogenicity, they may not fully reflect functional immunity or clinical protection. Another limitation relates to the interval-censored nature of the immunogenicity data, which may have resulted in the Kaplan-Meier approach overestimating survival relative to the true, unobserved event times.

Because of the limited number of events, adjustment in the multivariable Cox model for factors associated with global response was restricted to age and BMI. Therefore, residual confounding from other unmeasured or unadjusted factors cannot be excluded, and the adjusted estimates should be interpreted with caution. Given the limited sample size, our study may have been underpowered to detect modest differences between groups, particularly in serotype-specific analyses. Lastly, no adjustment for multiple comparisons was performed because of the exploratory nature of these analyses and the limited sample size. Consequently, multiple testing across serotypes, endpoints, and time points may have increased the risk of chance findings.

In conclusion, the serological response following a pneumococcal vaccination schedule consisting of one dose of PCV13 followed 2 months later by one dose of PPV23 was heterogeneous across serotypes and appeared generally higher in DP than in KTR. The response was particularly modest when assessed using the stringent combined endpoint of both seroconversion and seroprotection for at least five serotypes. Although immunogenicity provides useful information on vaccine-induced immune responses, helping to identify populations with suboptimal vaccine-induced immunity and inform future vaccination strategies, further studies are needed to determine how these responses translate into protection against pneumococcal disease. In addition, as higher-valent conjugate vaccines such as PCV20 become increasingly incorporated into pneumococcal vaccination strategies, comparative studies will be important to determine the optimal vaccination approach in immunocompromised populations.

## Data Availability

The original contributions presented in the study are included in the article/supplementary material, further inquiries can be directed to the corresponding author/s.

## Supplementary material

Supplementary Figure 1 Flowchart of study participants.

Supplementary Figure 2 Sensitivity analysis of time to seroconversion using the Turnbull estimator accounting for interval censoring, by pneumococcal serotype. P values correspond to the generalized log-rank test for interval-censored data.

Supplementary Table 1 Estimated slopes (β), 95% confidence intervals for each segment of the final piecewise linear mixed-effects models. Log_10_-transformed IgG levels over time were modelled using a piecewise linear mixed-effects model. The best-fitting model included a significant change in slope at month 2.

Supplementary Table 2 Factors associated with global response. Estimates were made from univariable and multivariable Cox models. Predictors in the multivariable model were selected based on clinical relevance and model parsimony.

## References

[1] Krueger KM Ison MG Ghossein C . Practical guide to vaccination in all stages of CKD, including patients treated by dialysis or kidney transplantation. Am J Kidney Dis. (2020) 75:417–25. doi: 10.1053/j.ajkd.2019.06.014 31585683

[2] Slinin Y Foley RN Collins AJ . Clinical epidemiology of pneumonia in hemodialysis patients: the USRDS waves 1, 3, and 4 study. Kidney Int. (2006) 70:1135–41. doi: 10.1038/sj.ki.5001714 16871243

[3] Vandecasteele SJ Ombelet S Blumental S Peetermans WE . The ABC of pneumococcal infections and vaccination in patients with chronic kidney disease. Clin Kidney J. (2015) 8:318–24. doi: 10.1093/ckj/sfv030 26034594 PMC4440476

[4] Marques Antunes M Duarte GS Brito D Borges M Costa J Ferreira JJ . Pneumococcal vaccination in adults at very high risk or with established cardiovascular disease: systematic review and meta-analysis. Eur Heart J Qual Care Clin Outcomes. (2021) 7:97–106. doi: 10.1093/ehjqcco/qcaa030 32259237

[5] Pierre É Pladys A Bayat-Makoei S Tattevin P Vigneau C . Pneumococcal vaccination coverage at the initiation of chronic dialysis, and its association with mortality during the first year. Vaccine. (2023) 41:3655–62. doi: 10.1016/j.vaccine.2023.05.003 37179164

[6] Yamaguchi K Kitamura M Takazono T Hashiguchi J Funakoshi S Mukae H . Prognoses of patients undergoing hemodialysis administered 23-valent pneumococcal polysaccharide versus 13-valent pneumococcal protein conjugate vaccines. J Infect Chemother. (2023) 29:1126–31. doi: 10.1016/j.jiac.2023.08.010 37604429

[7] Ihara H Kikuchi K Taniguchi H Fujita S Tsuruta Y Kato M . 23-valent pneumococcal polysaccharide vaccine improves survival in dialysis patients by preventing cardiac events. Vaccine. (2019) 37:6447–53. doi: 10.1016/j.vaccine.2019.08.088 31526624

[8] Gilbertson DT Guo H Arneson TJ Collins AJ . The association of pneumococcal vaccination with hospitalization and mortality in hemodialysis patients. Nephrol Dial Transplant. (2011) 26:2934–9. doi: 10.1093/ndt/gfq853 21317410

[9] Drijkoningen JJ Rohde GG . Pneumococcal infection in adults: burden of disease. Clin Microbiol Infect. (2014) 20:45–51. doi: 10.1111/1469-0691.12461 24313448

[10] van der Poll T Opal SM . Pathogenesis, treatment, and prevention of pneumococcal pneumonia. Lancet. (2009) 374:1543–56. doi: 10.1016/S0140-6736(09)61114-4 19880020

[11] Garcia Garrido HM Schnyder JL Haydari B Vollaard AM Tanck MWT de Bree GJ . Immunogenicity of the 13-valent pneumococcal conjugate vaccine followed by the 23-valent pneumococcal polysaccharide vaccine in people living with HIV on combination antiretroviral therapy. Int J Antimicrob Agents. (2022) 60:106629. doi: 10.1016/j.ijantimicag.2022.106629 35760223

[12] Osmanodja B Ronicke S Budde K Jens A Hammett C Koch N . Serological response to three, four and five doses of SARS-CoV-2 vaccine in kidney transplant recipients. J Clin Med. (2022) 11:2565. doi: 10.3390/jcm11092565 35566691 PMC9105533

[13] Liefeldt L Glander P Klotsche J Straub-Hohenbleicher H Budde K Eberspächer B . Predictors of serological response to SARS-CoV-2 vaccination in kidney transplant patients: Baseline characteristics, immunosuppression, and the role of IMPDH monitoring. J Clin Med. (2022) 11:1697. doi: 10.3390/jcm11061697 35330022 PMC8953201

[14] Available online at: https://sante.gouv.fr/IMG/pdf/Calendrier_vaccinal_2015 (Accessed November 13, 2024).

[15] Robbins A Bahuaud M Hentzien M Maestraggi Q Barbe C Giusti D . The 13-valent pneumococcal conjugate vaccine elicits serological response and lasting protection in selected patients with primary humoral immunodeficiency. Front Immunol. (2021) 12:697128. doi: 10.3389/fimmu.2021.697128 34290713 PMC8287634

[16] Flem E Mouawad C Palmu AA Platt H Johnson KD McIntosh ED . Indirect protection in adults ≥18 years of age from pediatric pneumococcal vaccination: a review. Expert Rev Vaccines. (2024) 23(1):997ߝ1010. doi: 10.1080/14760584.2024.2416229 39435466

[17] Mahmoodi M Aghamohammadi A Rezaei N Lessan-Pezeshki M Pourmand G Mohagheghi MA . Antibody response to pneumococcal capsular polysaccharide vaccination in patients with chronic kidney disease. Eur Cytokine Netw. (2009) 20:69–74. doi: 10.1684/ecn.2009.0153 19541592

[18] Shahi S Tavana S Alirezaei A Sayadi L . Effect of pneumococcal conjugate 13 valent and polysaccharide 23 valent vaccination on anti-pneumococcal antibody titer of hemodialysis patients: a randomized clinical trial. Tanaffos. (2023) 22:143–51. PMC10618593 37920305

[19] Eriksson M Käyhty H Saha H Lahdenkari M Koskinen P Mäkisalo H . A randomized, controlled trial comparing the immunogenecity and safety of a 23-valent pneumococcal polysaccharide vaccination to a repeated dose 13-valent pneumococcal conjugate vaccination in kidney transplant recipients. Transpl Infect Dis. (2020) 22:e13343. doi: 10.1111/tid.13343 32473046

[20] Pourfarziani V Ramezani MB Taheri S Izadi M Einollahi B . Immunogenicity of pneumococcal vaccination in renal transplant recipients and hemodialysis patients: a comparative controlled trial. Ann Transplant. (2008) 13:43–7. 18806734

[21] Vandecasteele SJ De Bacquer D Caluwe R Ombelet S Van Vlem B . Immunogenicity and safety of the 13-valent pneumococcal conjugate vaccine in 23-valent pneumococcal polysaccharide vaccine-naive and pre-immunized patients under treatment with chronic haemodialysis: a longitudinal quasi-experimental phase IV study. Clin Microbiol Infect. (2018) 24:65–71. doi: 10.1016/j.cmi.2017.05.016 28559003

[22] Food and Drug Administration . PREVNAR 20 (Pneumococcal 20-Valentconjugate Vaccine) Prescribing Information (2021). Available online at: https://www.fda.gov/media/149987/download (Accessed 2023 September 20).

