# Supplementary material for: Immunogenicity of sequential conjugate and non-conjugate pneumococcal vaccination in dialysis patients and kidney transplant recipients: a 12-month serological follow-up
Source: Front Immunol. 2026 Oct 8;17:1906893. doi: 10.3389/fimmu.2026.1906893 (PMC13687303; doi:10.3389/fimmu.2026.1906893)
Supplement: Supplementary file 1 [file Supplementaryfile1.pdf]

Sup Fig 1: Flowchart of study participants

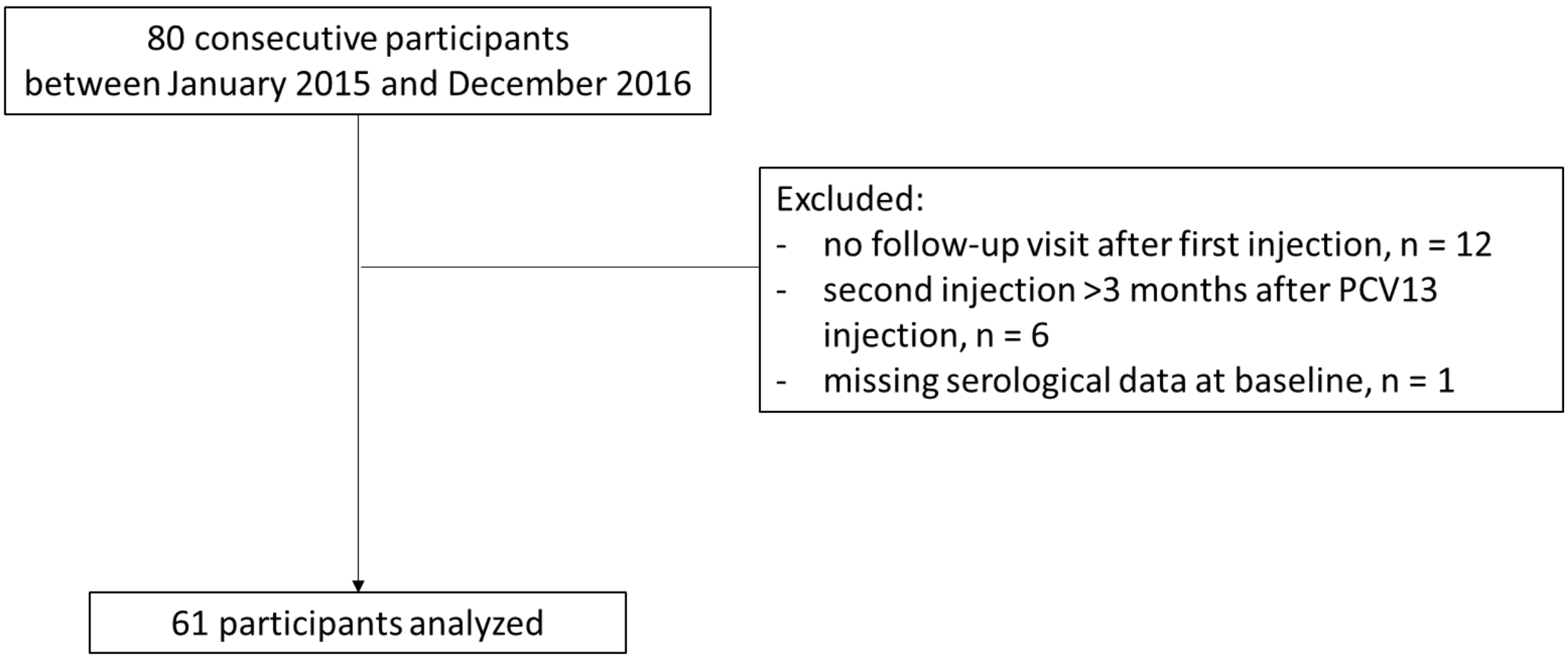

Sup Fig 2: Sensitivity analysis of time to seroconversion using the Turnbull estimator accounting for interval censoring, by pneumococcal serotype

P values correspond to the generalized log-rank test for interval-censored data

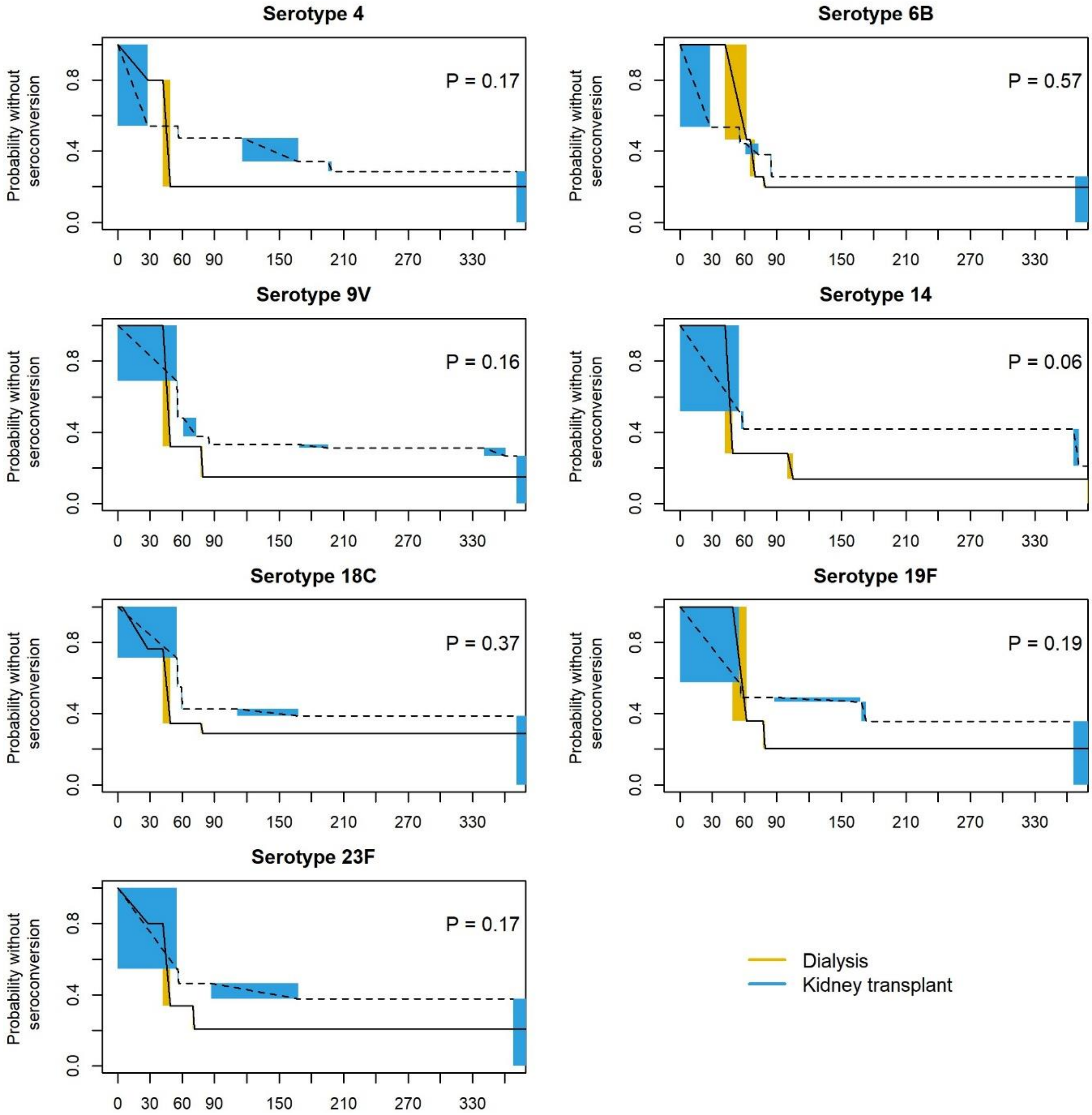

Sup Tab 1: Estimated slopes (β), 95% confidence intervals for each segment of the final piecewise linear mixed-effects models.

Log_{10}-transformed IgG levels over time were modelled using a piecewise linear mixed-effects model. The best-fitting model included **a significant change in slope at month 2.**

Serotype	Slopes (log µg/mL/month) by time interval	Dialysis patients (DP)	Kidney transplant recipients (KTR)
4	M0 – M2	0.4 (0.3 – 0.5)	0.3 (0.2 – 0.3)
	M2 – M12	-0.02 (-0.04 – 0.007)	-0.02 (-0.04 – 0.004)
6B	M0 – M2	0.4 (0.3 – 0.5)	0.3 (0.2 – 0.4)
	M2 – M12	-0.02 (-0.05 – 0.006)	-0.02 (-0.04 – 0.003)
9V	M0 – M2	0.3 (0.3 – 0.4)	0.2 (0.2 – 0.3)
	M2 – M12	-0.01 (-0.04 – 0.009)	-0.02 (-0.04 – -0.001)
14	M0 – M2	0.4 (0.3 – 0.5)	0.2 (0.1 – 0.3)
	M2 – M12	-0.006 (-0.03 – 0.02)	-0.009 (-0.03 – 0.01)
18C	M0 – M2	0.4 (0.3 – 0.5)	0.3 (0.2 – 0.3)
	M2 – M12	-0.02 (-0.05 – 0.003)	-0.02 (-0.04 – 0.003)
19F	M0 – M2	0.3 (0.2 – 0.4)	0.3 (0.2 – 0.4)
	M2 – M12	-0.02 (-0.04 – 0.007)	-0.02 (-0.04 – -0.0008)
23F	M0 – M2	0.4 (0.3 – 0.6)	0.3 (0.2 – 0.4)
	M2 – M12	-0.02 (-0.05 – 0.01)	-0.02 (-0.05 – 0.003)

Sup Tab 2. Factors associated with global response

Estimates were made from univariable and multivariable Cox models. Predictors in the multivariable model were selected based on clinical relevance and model parsimony.

	Univariable				Multivariable[1]		
**Characteristic**	**N**	**HR**	**95% CI**	**P-value**	**HR**	**95% CI**	**P-value**
Kidney transplant vs. Dialysis	61						
0 – 3 months		0.51	(0.23 – 1.11)	0.09	0.53	(0.23 – 1.17)	0.11
>3 months		0.27	(0.05 – 1.62)	0.15	0.18	(0.02 – 1.15)	0.07
Age, years	61	0.98	(0.96 – 1.00)	0.10	0.98	(0.97 – 1.01)	0.20
Body mass index,							
Abnormal vs normal (18.5-25 kg/m²)	60	0.38	(0.18 – 0.83)	0.01	0.36	(0.16 – 0.80)	0.01
Sex,							
Male vs Female	61	0.78	(0.37 – 1.65)	0.52			
Diabetes	61	0.69	(0.32 – 1.50)	0.35			

[1] N = 60 individuals included in the analysis and N = 33 events

Abbreviations: CI, confidence interval; HR, hazard ratio
